# Three and a Half Decades of Pediatric Heart Transplantation: Evolution of Surgical Practice and Outcomes at a High-Volume Centre

**DOI:** 10.3390/jcdd13060267

**Published:** 2026-06-12

**Authors:** Mohamed Salem, Martin Leroy, Thomas Zajons, Mohammed Al-Tawil, Assad Haneya, Susanne Skrzypek, Joseph Thul, Matthias Müller, Christian Jux, Hakan Akintürk

**Affiliations:** 1 Department of Congenital Heart Surgery, Pediatric Heart Centre, University Hospital of Giessen and Marburg, Justus Liebig University, 35390 Gießen, Germany; 2 Centre for Congenital Heart Disease/Pediatric Cardiology, Heart and Diabetes Centre NRW, University Clinic of Ruhr-University Bochum, 32545 Bad Oeynhausen, Germany; 3 Department Pediatric Cardiac Anesthesia, Pediatric Heart Centre, University Hospital of Giessen and Marburg, Justus Liebig University, 35390 Gießen, Germany; 4 Department of Cardiovascular Surgery, Krankenhaus der Barmherzigen Brüder Trier, 54942 Trier, Germany; 5 Department of Pediatric Cardiology, Pediatric Heart Centre, University Hospital of Giessen and Marburg, Justus Liebig University, 35390 Gießen, Germany

**Keywords:** pediatric heart transplantation, evolution of pediatric heart transplantaion, congenital heart defects

## Abstract

Background: Heart transplantation (HTx) is a well-established therapy for pediatric patients with end-stage heart failure. Over the past decades, the field has considerably evolved, with noticeable changes in surgical techniques and post-transplant outcomes. This study presents our center’s experience over the past three decades. Methods: Between 1988 and 2024, we performed 256 heart transplants in pediatric patients (<18 years) with congenital heart defects (CHD) or myopathy. We divided our cohort into three periode, eras: Era1 (1988–1999), Era2 (2000–2011), and Era3 (2012–2024). We analyzed and reported baseline patient data, postoperative outcomes, and survival analysis. Results: In the first era, most HTx recipients were infants (75%), with CHD accounting for 75% of cases. In the latest era, older children and adolescents were transplanted more frequently with infants representing only 22%, and myopathies became a more predominant indication, representing 57% of patients. The use of mechanical circulatory support increased significantly (<0.001), and a complete shift towards the bi-caval surgical technique was achieved in the recent era. In terms of post-HTx outcomes, 30-day mortality and allograft vasculopathy significantly decreased in the recent era compared with previous periods (<0.001). Conversely, operative time and post-HTx hemodialysis were more frequently observed in the recent era (<0.001). Long-term survival numerically improved in the middle and recent eras compared with the early era; however, no statistically significant difference in Kaplan–Meier survival across eras was observed (log-rank *p* = 0.19). Conclusions: Over the past three decades, HTx in pediatric patients has evolved, with improvements in early survival and reduced allograft vasculopathy. Changes in patient demographics, surgical technique, and use of MCS in the recent era highlight the ongoing progress as well as the remaining challenges in this complex population.

## 1. Introduction

Heart transplantation (HTx) remains the definitive therapy for children with end-stage heart failure resulting from congenital heart defects (CHD) or cardiomyopathies. Since the first successful pediatric HTx in 1984, continuous advancements in surgical techniques, perioperative care, and immunosuppression have markedly improved outcomes [1,2].

Over the past two decades, the International Society for Heart and Lung Transplantation (ISHLT) has reported measurable milestones, demonstrating significant changes in global HTx practices for both pediatric and adult populations [2,3]. These developments have influenced patient selection, pre- and postoperative management, and the utilization of durable mechanical circulatory support (MCS) [3].

Compared to adult HTx, pediatric cases are inherently more complex and resource-intensive, often involving patients with complex CHD who have undergone multiple prior palliative procedures [4]. Additionally, factors such as donor organ availability and size matching continue to limit timely transplantation in this population [3,5].

Despite improvements in pre-transplant evaluation, patient selection, and the use of MCS, long-term outcomes after pediatric HTx remain limited by rejection, allograft vasculopathy, and graft failure. Contemporarily, up to 10–25% of patients are treated for rejection during the first year after transplant [6,7].

This study aims to evaluate the temporal trends in pediatric HTx at our institution over the past three decades, focusing on changes in characteristics of the patient population, operative practices, and post-transplant outcomes. We aim to highlight areas of progress as well as persistent challenges in the care of this complex population.

## 2. Materials and Methods

### 2.1. Data Acquisition and Study Design

We retrieved the data of 256 heart transplants that were performed between 1988 and 2024 in our center for patients with CHD or cardiomyopathy. All included patients were younger than <18 years old at the time of transplantation. Patients who were re-transplanted were excluded. Patients who died on the waiting list for HTx or those still listed for HTx were also excluded from the analysis. All donor organs were retrieved from patients with confirmed brain death. Patients undergoing re-transplantation as the initial transplantation procedure were excluded from the study cohort. Patients requiring re-transplantation during follow-up after primary pediatric HTx remained included in the survival analyses.

### 2.2. Definitions

Patients were classified as having CHD or cardiomyopathy as the primary indication for HTx. CHD included all structural congenital malformations of the heart or great vessels requiring surgical or palliative intervention, such as hypoplastic left heart syndrome (HLHS), double-inlet ventricle, transposition of the great arteries (TGA), imbalanced atrioventricular septal defect (AVSD), and other single-ventricle or complex biventricular lesions.

Cardiomyopathy encompassed dilated, restrictive, hypertrophic, or non-compaction cardiomyopathies, as well as myocarditis-related or chemotherapy-induced myocardial dysfunction. Patients with both congenital defects and myocardial abnormalities were classified according to the predominant pathophysiologic substrate prompting HTx.

Acute rejection was defined as biopsy-proven cellular or antibody-mediated rejection (ISHLT grade ≥ 1R) or clinically suspected rejection requiring intensification of immunosuppression. Graft vasculopathy (cardiac allograft vasculopathy, CAV) is defined as angiographic or intravascular ultrasound evidence of new or progressive coronary narrowing, graded according to ISHLT criteria.

Chronic kidney disease (CKD) was defined as persistent renal dysfunction documented during follow-up, including reduced estimated glomerular filtration rate and/or the need for chronic renal replacement therapy according to the treating multidisciplinary team assessment. Graft dysfunction was defined as clinically relevant ventricular dysfunction of the transplanted heart associated with impaired cardiac output requiring inotropic, mechanical circulatory, or intensive care support in the absence of acute rejection or surgical complications. High systolic transpulmonary gradient was defined as elevated transpulmonary pressure gradient on cardiac catheterization or echocardiographic assessment despite optimized medical therapy. Uncontrolled infection was defined as severe bacterial, viral, or fungal infection resulting in sepsis, multiorgan dysfunction, prolonged intensive care treatment, or death despite anti-infective therapy. Postoperative neurological complications included ischemic or hemorrhagic stroke, hypoxic–ischemic brain injury, or clinically relevant seizures occurring during the postoperative hospitalization period. Postoperative bleeding included clinically significant intraoperative or postoperative bleeding requiring surgical revision, transfusion support, or invasive intervention.

In terms of era classification, patients were divided into three eras based on the year of transplantation: Era 1 (1988–1999), Era 2 (2000–2011), and Era 3 (2012–2024). Accordingly, we reported and contrasted temporal changes in baseline patient profiles, operative details, and postoperative and long-term outcomes.

### 2.3. Statistical Analysis

Baseline patient characteristics, operative variables, and postoperative outcomes were summarized using descriptive statistics. The distribution of continuous variables was assessed with the Shapiro–Wilk test to determine normality. “Depending on data distribution, comparisons between groups were made using one-way ANOVA for normally distributed variables or the Kruskal–Wallis test for non-normally distributed variables. Continuous data are presented as medians with interquartile ranges (IQRs).” Categorical variables were expressed as counts (n) and percentages (%) and compared using the chi-square or Fisher’s exact test, as appropriate. Survival analyses were performed using the Kaplan–Meier method, and survival differences between groups were evaluated using the log-rank test. Designated year-specific survival probabilities were also provided accordingly. All analyses were conducted using R Software (Version 4.5.1).

### 2.4. Surgical Methods

Surgical techniques evolved substantially over time, with a transition from predominantly bi-atrial to bi-caval implantation. Complex anatomies, including failing Fontan and heterotaxy syndromes, required individualized reconstruction strategies as previously described. Detailed surgical techniques have been known and are beyond the scope of this manuscript.

## 3. Results

### 3.1. Overall Description of Included Patients

A total of 256 patients were included in the analysis, of whom 107 (42%) were infants. Male patients comprised 150 (59%) of the cohort. The median age at the time of transplantation was 1.6 years (interquartile range [IQR]: 0.4–8.9 years). Overall, 139 patients (54%) underwent HTx for CHD, whereas 117 (46%) underwent HTx for cardiomyopathy. Regarding transplant era, 77 patients (30%) underwent HTx in the early era (1988–1999), 88 (34%) in the middle era (2000–2011), and 91 (36%) in the recent era (2012–2024).

### 3.2. Temporal Trends in Patients’ Profiles

**Figure 1:**
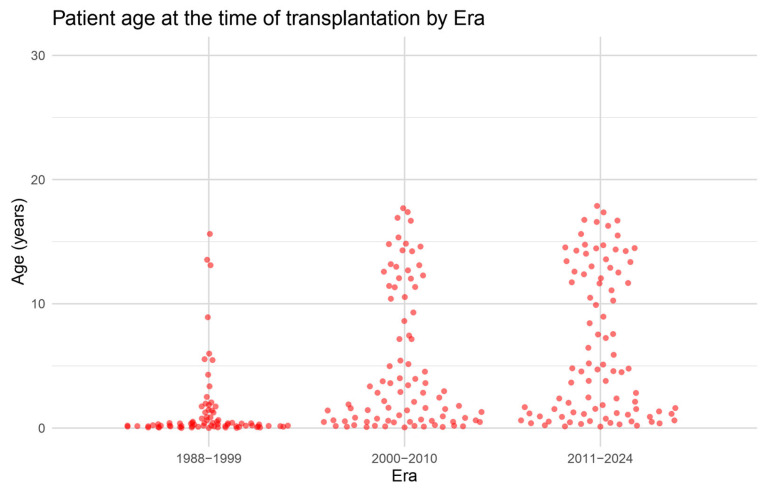
Patient age (under 18 years old) per transplantation era.

Over the past three decades, there has been a notable shift toward the inclusion of older pediatric patients, accompanied by a decline in infant transplants. In the early era, infants (<1 year) represented 75% of the HTx population at our center, compared with only 22% in the most recent era (*p* < 0.001) (Figure 1).

In terms of primary indication for HTx, CHD accounted for the majority of transplants in the early era (77%), where univentricle heart represents 62% and HLHS 57%. Over time, there was a steady shift toward cardiomyopathy as a more frequent indication. In our cohort, 48 patients with HLHS underwent primary heart transplantation before 2000, without prior staged palliation. In contrast, later HLHS patients more frequently underwent transplantation after staged palliation, including after Stage I in 16 patients, after Stage II in 14 patients, and after TCPC/Fontan completion in 25 patients. In the recent era, cardiomyopathy represented a slightly higher proportion of patients undergoing HTx compared with CHD (57% vs. 43%, respectively). There was a steady increase in the use of palliative surgeries prior to HTx across eras. Patients with a classic Glenn shunt at the time of HTx represented only 1.3% of the cohort in the early era but this increased to 23% in the recent era. Similarly, patients with a Fontan circulation rose from 1.3% in the early era to 15% in the most recent era.

In the latest era, HTx in ABO-incompatible patients was introduced, accounting for 9.9% of all transplants performed during this period. The utilization of cytomegalovirus (CMV)-positive donor hearts for CMV-negative recipients increased markedly over time, rising from 15% in the early era to 31% in the recent era.

**Figure 2:**
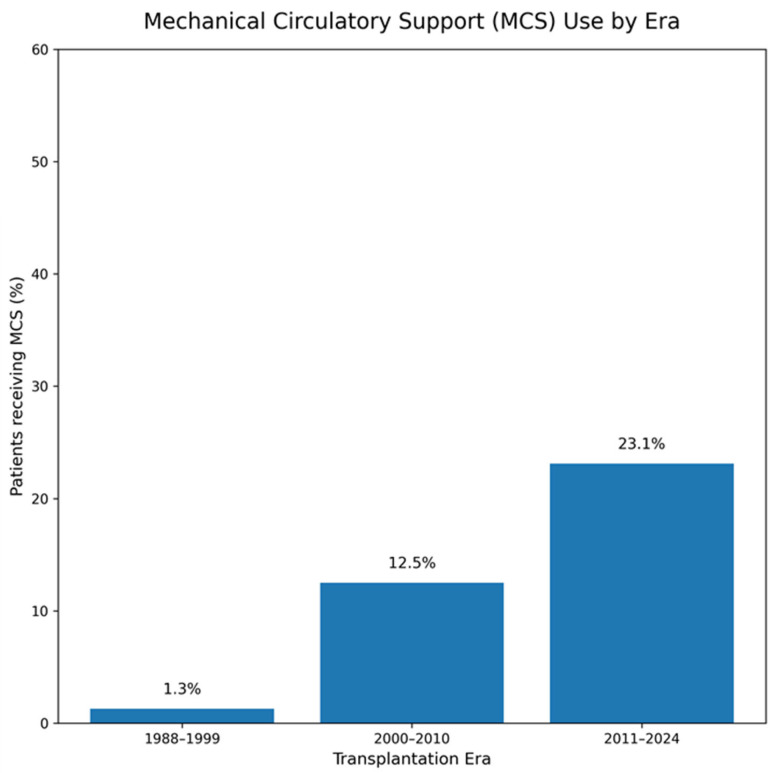
Temporal use of MCS support.

The use of MCS increased steadily across eras. At our center, 23% of patients who underwent HTx in the recent era were supported with MCS at baseline prior to heart transplantation, compared with only 1.3% in the early era and 13% in the middle era. There was no consistent trend in MCS duration across eras, as shown in Figure 2.

In total, 31 patients underwent mechanical circulatory support (MCS) prior to heart transplantation. Of these, 17 patients received LVAD support, including 16 Berlin Heart EXCOR systems and one MEDOS system, while 13 patients required BiVAD support, including 12 Berlin Heart EXCOR systems and one MEDOS system. Only one patient underwent transplantation directly under VA-ECMO support. However, temporary VA-ECMO was used more frequently as an initial stabilization strategy prior to conversion to durable ventricular assist devices. Specifically, three patients were transitioned from VA-ECMO to LVAD support and four patients from VA-ECMO to BiVAD support before transplantation. This reflects our institutional strategy to preferentially bridge patients from temporary extracorporeal support to durable MCS whenever feasible prior to transplantation.

However, the overall waitlist time after listing for HTx increased significantly, from a median of 1.5 months (IQR: 0.6–2.2) in the early era to 2.5 months (IQR: 0.7–6.6) in the recent era. Table 1 summarizes the temporal changes in patients’ profiles across the three defined eras.

### 3.3. Temporal Trends in Surgical Practice

**Figure 3:**
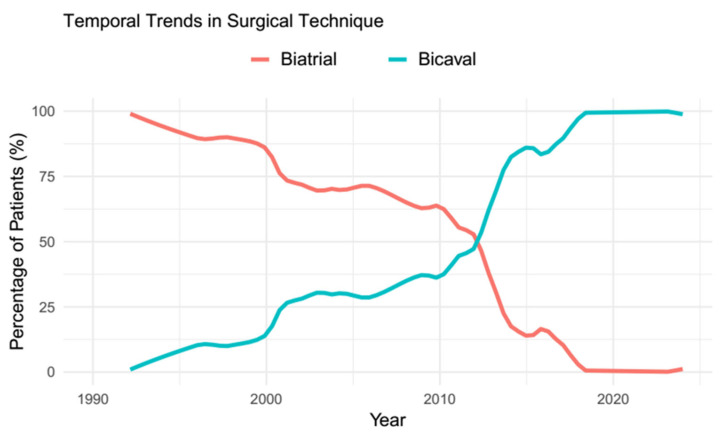
Temporal trend of the use of bi-caval and bi-atrial surgical techniques (Cochran–Armitage trend *p* < 0.001).

There was a clear and steady shift in surgical practice at our center over time, from predominantly bi-atrial anastomosis in the early era (89%) to widespread adoption of the bi-caval technique in the most recent era (89%). The proportion of bi-caval transplants increased from 11% in the early era to 89% in the recent era (*p* < 0.001), as shown in Figure 3.

Both donor ischemic time and operative time were longest in the most recent era compared with the previous two eras due to the higher complexity of redo surgery. The middle era demonstrated the shortest donor ischemic and cardiopulmonary bypass times (*p* < 0.001). Table 2 summarizes operative characteristics across eras, while Figure 3 illustrates the progressive shift toward adoption of the bi-caval technique for heart transplantation.

### 3.4. Temporal Trends in HTx Outcomes

There was a notable increase in the proportion of patients requiring post-HTx hemodialysis in the recent era (20%), compared with 5.7% in the preceding era. In contrast, the prevalence of persistent kidney disease declined markedly over time, from 80% in the early era to 28% in the most recent era, as shown in Table 3.

The rates of bleeding and neurological complications (ischemic or hemorrhagic stroke, hypoxic–ischemic brain injury or seizures) remained stable across eras, with no significant temporal trends. The incidence of acute rejection showed a modest decrease, from 68% in the early era to 54% in the most recent era.

Graft vasculopathy was more commonly observed among patients who underwent HTx in earlier eras, being documented in 45%, 35%, and 11% of patients in the early, middle, and recent eras, respectively. However, earlier recipients have documented longer follow-up times. In the recent era, most patients (89%) had non-significant graft vasculopathy, whereas in the earliest era, 50% had mild disease and 21% had severe vasculopathy or graft dysfunction. Moreover, infant recipients showed lower rates of cardiac allograft vasculopathy compared with older pediatric recipients. Among transplanted infants with HLHS, the median time to CAV was significantly longer than in other infants with congenital heart disease who had undergone prior surgical palliation or multiple surgical procedures (19 years vs. 9 years, *p* = 0.004).

Permanent pacemaker implantation remained relatively uncommon across all eras, occurring in 2.5%, 4.7%, and 3.5% of patients, respectively.

The elevated pulmonary vascular resistance was observed more frequently in recent transplants (29% of patients), and the use of postoperative ECMO/ECLS also increased in the last two eras (17% and 13%).

Thirty-day mortality decreased substantially over time, from 13% in the early era to 2.3% and 2.2% in the middle and recent eras, respectively. Re-transplantation (re-HTx) was required in seven patients (9.1%) from the first era, two patients from the middle era, and one patient in the most recent era.

### 3.5. Long-Term Survival

**Figure 4:**
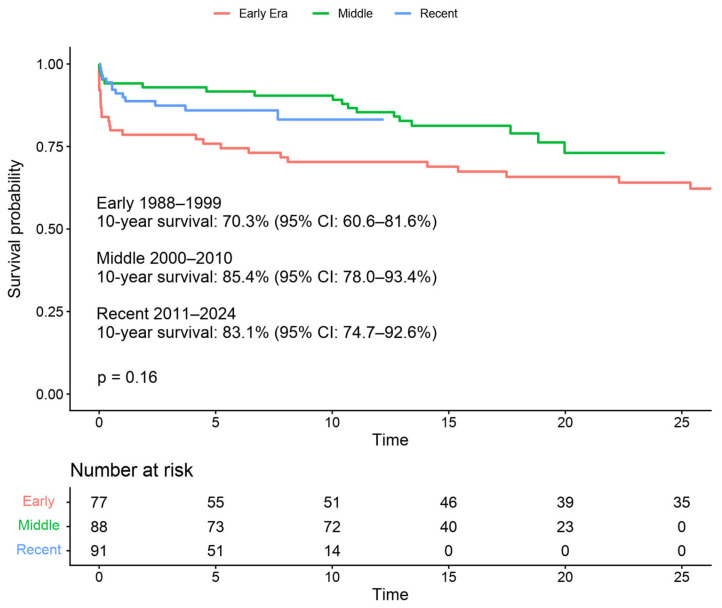
Survival per era.

Kaplan–Meier analysis demonstrated numerically improved long-term survival in the middle and recent eras compared with the early era. The 10-year survival rates were 69.7%, 87.8%, and 84.1% for the early, middle, and recent eras, respectively. However, the overall difference in long-term survival across eras did not reach statistical significance (log-rank *p* = 0.16) (Figure 4).

**Figure 5:**
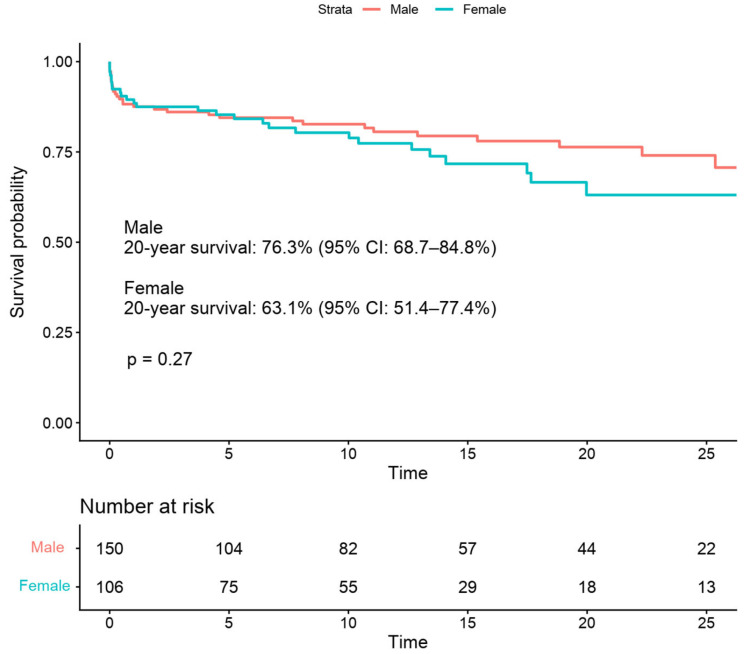
Survival per recipient’s sex.

Female recipients demonstrated lower overall survival compared with male recipients at 20 years of follow-up; however, this difference did not reach statistical significance (76.3% vs. 63.1%, *p* = 0.27), as shown in Figure 5.

**Figure 6:**
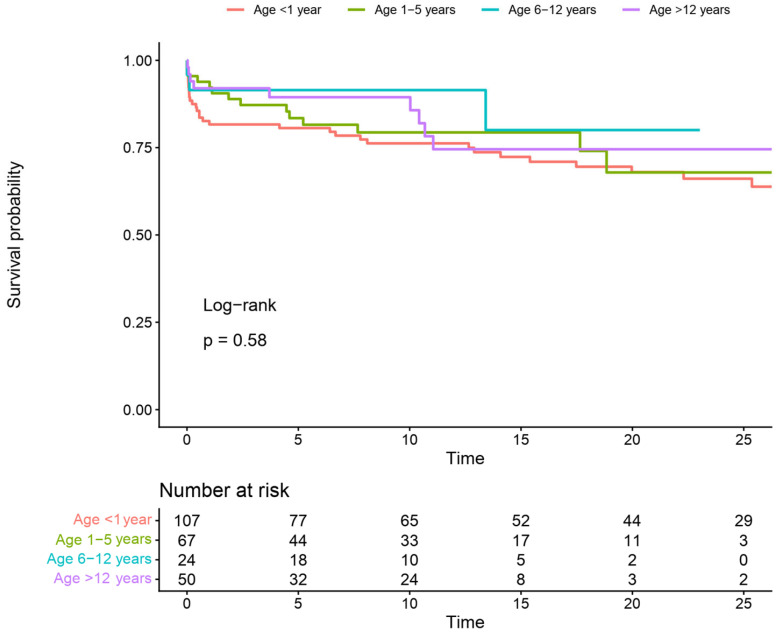
Survival per age group at the time of transplantation.

Similarly, infant recipients (<1 year of age) demonstrated lower long-term survival compared with older pediatric recipients (≥1 year of age), although this difference did not reach statistical significance (20-year survival: 67.9% vs. 72.8%, respectively; log-rank *p* = 0.14) (Figure 6).

## 4. Discussion

Our single-center study provides a comprehensive overview of pediatric HTx over three and a half decades, highlighting temporal changes in patient characteristics, surgical practice, and outcomes. Across our defined eras (1988–1999, 2000–2011, and 2012–2024), we observed significant evolution in patient selection, operative details, and postoperative outcomes. The following table highlights the major era-related changes in patient characteristics, surgical strategies, perioperative management, and outcomes, together with their likely clinical and institutional drivers (Table 4). 

In terms of patients’ demographics and pre-transplant profiles, there has been a clear shift in both recipient age and the primary indication for HTx. While CHD was the predominant indication in earlier years, cardiomyopathy has become more frequent in recent eras [8,9]. In our cohort, 57% in the first era was primary HLHS without palliative surgery. The high proportion of infants in the early era was largely driven by the historical referral pattern for HLHS at our center. Before 2000, many patients with HLHS underwent primary neonatal or infant heart transplantation without prior staged palliation. In later eras, transplantation was more commonly performed after previous surgical palliation, including after Stage I, Stage II, or TCPC/Fontan completion. This shift reflects the broader evolution of HLHS management from primary transplantation toward staged surgical palliation and explains the marked decline in infant transplantation over time. This trend is consistent with international registry data, particularly within European cohorts. According to the 2023 ISHLT Pediatric Heart Transplant Registry Report, regional differences persist in the distribution of transplant indications. In Europe, more than half of pediatric recipients underwent HTx for dilated cardiomyopathy, whereas only about one quarter had CHD as the underlying diagnosis. In contrast, in North America, cardiomyopathy and CHD remain nearly equally represented, each accounting for approximately 40% of pediatric transplants. In other world regions, cardiomyopathy predominates, representing over 70% of cases, with CHD contributing to fewer than 10%. These geographic variations are influenced by the higher proportion of infant transplants in North America—where CHD is the most common diagnosis—as well as by regional differences in donor availability and the clinical management of complex CHD [9].

The introduction of staged palliation (e.g., Glenn and Fontan procedures) and the lack of infant suitable donors as well as the variable regional referral rates may together explain the declining number of HTx in infants with CHD [9,10,11,12].

Taken together, the temporal shift observed at our center parallels these international trends, reflecting evolving case selection and transplant candidacy criteria in pediatric populations worldwide.

On a positive note, the introduction of ABO-incompatible HTx at our center reflects growing center capability and more efficient donor utilization. Similarly, the greater use of CMV-positive donors for CMV-negative recipients parallels global practice trends considering improved antiviral prophylaxis and monitoring [13].

The adoption of MCS as a bridge to HTx has risen steadily, mirroring international experience with durable ventricular assist devices in children. Data from the Paedi-EUROMACS Registry, which reported 398 implants in 353 pediatric patients, demonstrated a 1-year survival rate of 81.5% to the endpoints of transplantation, recovery, or ongoing device support. Furthermore, over 45% of patients were successfully transplanted within one year of MCS initiation [14]. This regional improvement in MCS outcomes has contributed to greater utilization and stability among pediatric candidates awaiting transplantation, but it also underscores the limited transplant rates among European centers. Namely, the PediMACS registry in North America reported that 67% of children were successfully bridged to HTx within 12 months of VAD implantation [15]. In our cohort, 31 patients required MCS before heart transplantation: 17 received LVAD support, 13 received BiVAD support, and only one patient was transplanted directly from VA-ECMO. However, VA-ECMO was used as initial stabilization in seven patients before conversion to durable VAD support: three to LVAD and four to BiVAD. This reflects our institutional preference to convert suitable patients from temporary VA-ECMO to durable MCS before transplantation.

Despite these advances, our analysis highlights an increase in donor ischemic and operative times in the most recent era. In our opinion, this likely reflects the surgical complexity of contemporary cases, including redo operations, transplantation following prior single or multiple palliative procedures, and recipients bridged with MCS. These factors contribute to a longer operative duration while reflecting the inclusion of high-risk and surgically challenging patients in the modern pediatric HTx era.

Similarly, the higher rates of postoperative hemodialysis observed in the recent era may be explained by the same factors, particularly prolonged bypass times [16,17]. Lipman and colleagues reported that approximately 66% of patients developed some degree of acute kidney injury following HTx, with 7% requiring dialysis. Longer bypass time was identified as an independent predictor of postoperative hemodialysis [17].

In our experience, acute rejection rates decreased modestly, consistent with refinement of immunosuppressive regimens. CAV was observed more frequently among earlier recipients; however, this finding should be interpreted cautiously because follow-up duration was longer in earlier eras and shorter in recent recipients. Nonetheless, most recipients in the recent era showed no or only mild CAV during available follow-up. This observation may also partly reflect historical practice in the early era, when many infants with HLHS underwent primary heart transplantation rather than staged palliative repair.

The increased use of postoperative ECMO/ECLS likely reflects the availability of advanced MCS to support a better recovery. It is typically reserved for patients with early graft dysfunction or severe perioperative compromise. Although it was linked to significantly inferior survival in the first year post-HTx, long-term outcomes among survivors are similar to patients who had no MCS [18]. This supports the judicious use of post-HTx ECMO/ECLS as a life-saving measure for patients in need. In our cohort there were no significant differences between pHTX with or without MCS.

Permanent pacemaker implantation remained relatively uncommon across all eras, occurring in 2.5%, 4.7%, and 3.5% of patients, respectively. Although statistical signifi-cance was observed, the absolute event rates remained low. This may reflect evolving sur-gical complexity, including a higher proportion of redo procedures and congenital heart disease with altered atrial anatomy in later eras.

Thirty-day mortality improved significantly from 13% in the early era to approximately 2% in the following two eras demonstrating significant progress in perioperative care.

Long-term survival following pediatric HTx at our center remains excellent with noticeable improvement in the last two eras. This is comparable to international registers with 10-year survival averaging between 75 and 85% [8,19,20]. Although long-term survival numerically improved in the middle and recent eras, no statistically significant difference in Kaplan–Meier survival across eras was observed. Nevertheless, the persistently high long-term survival rates in the contemporary era likely reflect advances in perioperative management, immunosuppression, and mechanical circulatory support strategies.

## 5. Strengths and Limitations

Our study’s major strength lies in its large single-center cohort spanning over three decades of pediatric HTx, allowing consistent evaluation of temporal trends in patient selection, surgical practice, and outcomes under a uniform institutional protocol. However, as a retrospective single-center analysis, the study is subject to inherent selection bias, and certain era-based differences in patient population and follow-up duration have probably influenced long-term outcome comparisons. Despite these limitations, the comprehensive clinical and operative data provide valuable insight into the evolution of pediatric HTx.

## 6. Conclusions

Over three decades, pediatric HTx at our center has evolved substantially, accompanied by greater use of MCS and refined surgical practices. Despite increasing procedural complexity, early and long-term survival outcomes have improved and remained stable during the past 20 years. Ongoing refinement of donor utilization to optimize transplantation rates, together with improved management of high-risk recipients, remains essential to sustain progress in modern pediatric HTx.

## Figures and Tables

**Table 1:** Baseline characteristics of transplant recipients.

Characteristic	Early Era (1988–1999) N = 77 ^1^	Middle Era (2000–2011) N = 88 ^1^	Recent Era (2012–2024) N = 91 ^1^	*p*-Value ^2^
Male sex	45 (58%)	54 (61%)	51 (56%)	0.8
Age	0.3 (0.1, 0.9)	2.8 (0.6, 11.3)	4.7 (1.1, 12.9)	<0.001
Infant (<1 year)	58 (75%)	29 (33%)	20 (22%)	<0.001
Pediatric (>1 years)	19 (25%)	59 (67%)	71 (78%)	<0.001
Weight	4 (4, 8)	11 (6, 32)	15 (7, 36)	<0.001
Height	56 (53, 78)	86 (60, 135)	99 (70, 153)	<0.001
BMI	13.30 (12.00, 14.24)	14.61 (13.22, 16.56)	14.88 (13.37, 16.49)	<0.001
Main diagnosis				<0.001
CHD	59 (77%)	41 (47%)	39 (43%)	
Cardiomyopathy	18 (23%)	47 (53%)	52 (57%)	
Univentricular heart	48 (62%)	28 (32%)	30 (33%)	<0.001
Hypoplastic left heart	43 (57%)	23 (26%)	22 (24%)	<0.001
ABO incompatible	0 (0%)	0 (0%)	9 (9.9%)	<0.001
Bilateral SVC	1 (1.3%)	1 (1.1%)	3 (3.3%)	0.6
L-TGA	2 (2.6%)	3 (3.4%)	2 (2.2%)	0.9
D-TGA	3 (3.9%)	4 (4.5%)	11 (12%)	0.062
LVOT lesion	46 (60%)	38 (43%)	30 (33%)	0.002
Complete AV defect	5 (6.5%)	4 (4.5%)	4 (4.4%)	0.8
Other biventricular CHD lesions	24 (31%)	20 (23%)	17 (19%)	0.2
DORV	2 (2.6%)	2 (2.3%)	9 (9.9%)	0.046
Heterotaxy	1 (1.3%)	1 (1.1%)	3 (3.3%)	0.6
Dextrocardia	0 (0%)	2 (2.3%)	3 (3.3%)	0.4
Previous Fontan operation	1 (1.3%)	10 (11%)	14 (15%)	0.008
CMV+ donor, CMV− recipient	10 (15%)	12 (14%)	28 (31%)	0.010
Mechanical Circulatory Support as Bridging to HTx				
Any	2 (2.6%)	9 (10%)	20 (22%)	<0.001
LVAD	1 (1.3%)	5 (5.7%)	11 (12%)	0.018
Bi-VAD	1 (1.3%)	3 (4.1%)	9 (10.2%)	0.019
ECMO	0 (0%)	0 (0%)	1 (1%)	0.039
MCS time (days)	15 (15, 15)	69 (53, 281)	58 (7, 124)	0.3
Previous sternotomy/prior cardiac surgery	5 (6.5%)	10 (11%)	8 (8.8%)	0.5
Waitlist time (months)	1.5 (0.6, 2.2)	2.3 (0.7, 6.6)	2.5 (0.7, 6.6)	0.012

^1^ n (%); median (Q1, Q3). ^2^ Pearson’s chi-squared test; Kruskal–Wallis rank sum test; Fisher’s exact test.

**Table 2:** Operative details.

Characteristic	Early Era (1988–1999) N = 77 ^1^	Middle Era (2000–2011) N = 88 ^1^	Recent Era (2012–2024) N = 91 ^1^	*p*-Value ^2^
Surgical approach				
Bi-caval technique	4 (11%)	22 (30%)	79 (89%)	<0.001
Bi-atrial technique	31 (89%)	52 (70%)	10 (11%)	<0.001
Donor heart ischemic time (min)	246 (212, 285)	223 (158, 280)	255 (210, 314)	0.003
Donor heart ischemic time >240 min	28 (54%)	27 (35%)	46 (53%)	0.027
Bypass time (min)	261 (196, 334)	235 (182, 292)	294 (222, 328)	0.007
Reperfusion time (min)	110 (90, 172)	118 (89, 150)	146 (121, 190)	<0.001
Intraoperative AV block	2 (2.6%)	1 (1.1%)	8 (8.8%)	0.039
Intraoperative sick sinus	2 (2.6%)	1 (1.1%)	2 (2.2%)	0.9
Kidney Tx	3 (3.9%)	1 (1.1%)	1 (1.1%)	0.5

^1^ n (%); median (Q1, Q3). ^2^ Pearson’s chi-squared test; Kruskal–Wallis rank sum test; Fisher’s exact test.

**Table 3:** Post-transplantation outcomes.

Characteristic	Early Era (1988–1999) N = 77 ^1^	Middle Era (2000–2011) N = 88 ^1^	Recent Era (2012–2024) N = 91 ^1^	*p*-Value ^2^
Post-transplant lymphoproliferative disorder	12 (16%)	14 (16%)	10 (11%)	0.6
Redo sternotomy	6 (7.8%)	12 (14%)	15 (16%)	0.2
Neurological complications	7 (9.1%)	16 (18%)	15 (16%)	0.2
Hemodialysis	9 (12%)	5 (5.7%)	18 (20%)	0.017
Peritoneal dialysis	3 (3.9%)	5 (5.7%)	8 (8.8%)	0.4
Perioperative bleeding				
Any bleeding	10 (17%)	31 (38%)	32 (35%)	0.016
Intraoperative bleeding	4 (6.7%)	14 (17%)	19 (21%)	0.061
Postop cardiac bleeding	2 (3.3%)	14 (17%)	8 (8.8%)	0.024
Cranial bleeding	2 (3.3%)	4 (4.9%)	7 (7.7%)	0.6
Organ bleeding	1 (1.7%)	0 (0%)	4 (4.4%)	0.2
Acute rejection	52 (68%)	57 (65%)	49 (54%)	0.15
Rejection type				0.4
Humoral rejection	1 (2.1%)	4 (7.7%)	5 (11%)	
Cellular rejection	41 (87%)	40 (77%)	38 (81%)	
Humoral and cellular	5 (11%)	8 (15%)	4 (8.5%)	
Cardiac allograft vasculopathy	35 (45%)	31 (35%)	10 (11%)	<0.001
Time to vasculopathy (years)	17 (11, 25)	9 (4, 18)	7 (2, 9)	0.002
Grade of vasculopathy				<0.001
Non-significant	13 (28%)	16 (36%)	42 (82%)	
Mild disease	23 (50%)	15 (34%)	5 (9.8%)	
Severe	7 (15%)	9 (20%)	3 (5.9%)	
Graft dysfunction	3 (6.5%)	4 (9.1%)	1 (2.0%)	
Permanent pacemaker implantation	2 (2.5%)	4 (4.7%)	3.5 (3.5%)	0.007
Multiorgan dysfunction	5 (6.5%)	8 (9.1%)	7 (7.7%)	0.8
High systolic transpulmonary gradient	3 (5.3%)	9 (12%)	14 (16%)	0.13
Elevated irreversible PVR	0 (0%)	2 (2.3%)	4 (4.4%)	0.2
Chronic kidney disease	47 (80%)	45 (56%)	26 (29%)	<0.001
Hypertension	42 (71%)	70 (86%)	69 (77%)	0.079
Progressive RVF	7 (12%)	6 (7.7%)	3 (3.4%)	0.15
Progressive systemic LVF	8 (14%)	7 (9.0%)	3 (3.4%)	0.073
Incurable malignancy	3 (3.9%)	4 (4.5%)	1 (1.1%)	0.4
Uncontrolled infection	15 (19%)	6 (6.8%)	7 (7.7%)	0.016
Postoperative ECMO/ECLS	4 (5.2%)	15 (17%)	12 (13%)	0.062
Immunosuppressive therapy				
Everolimus	35 (60%)	45 (60%)	50 (56%)	0.8
Sirolimus	1 (1.8%)	0 (0%)	2 (2.2%)	0.5
Tacrolimus	38 (66%)	67 (88%)	75 (84%)	0.002
Cyclosporin	52 (88%)	49 (63%)	33 (37%)	<0.001
Azathioprine	53 (90%)	61 (78%)	47 (53%)	<0.001
Mycophenolate	41 (71%)	68 (88%)	38 (43%)	<0.001
Steroids	37 (63%)	53 (68%)	55 (62%)	0.8
Thirty-day mortality	10 (13%)	2 (2.3%)	2 (2.2%)	0.006
Re-heart transplantation	7 (9.1%)	2 (2.3%)	1 (1.1%)	0.031
Follow-up (years)	21 (4, 29)	15 (13, 20)	6 (2, 9)	<0.001

^1^ n (%); median (Q1, Q3). ^2^ Pearson’s chi-squared test; Kruskal–Wallis rank sum test; Fisher’s exact test.

**Table 4:** Summary of the temporal evolution of pediatric heart transplantation across the three study eras, highlighting major changes in recipient characteristics, surgical strategies, perioperative management, and clinical outcomes together with their likely contributing factors.

Era	Early Era (1988–1999)	Middle Era (2000–2011)	Recent Era (2012–2024)
Major Findings	High proportion of neonatal/infant HTx, predominantly HLHS and CHD, frequent primary transplantation, predominant bi-atrial technique	Increasing cardiomyopathy cases, transition toward bi-caval technique, increased use of staged palliation before HTx	Older recipients, increased MCS utilization, predominant bi-caval implantation, lower 30-day mortality and reduced CAV
Likely Clinical Drivers	Limited staged palliation strategies for HLHS, high neonatal referral volume, early transplant era surgical standards	Evolution of congenital heart surgery, improved perioperative management, broader HTx selection	Advances in MCS, improved donor utilization, refined immunosuppression, improved surgical and intensive care management

## Data Availability

The original contributions presented in this study are included in the article. Further inquiries can be directed to the corresponding author.
